# Clinical reasoning for acute dyspnoea: comparison between final-year medical students from discipline- and competency-based undergraduate programmes

**DOI:** 10.1186/s12909-020-02055-y

**Published:** 2020-05-19

**Authors:** Anja Czeskleba, Ylva Holzhausen, Harm Peters

**Affiliations:** Dieter Scheffner Centre for Medical Education and Educational Research, Dean’s Office of Study Affairs, Charité – Universitätsmedizin Berlin, Charité Platz 1, 10117 Berlin, Germany

**Keywords:** Clinical reasoning, Final-year clerkship students, Discipline-based medical education, Competency-based medical education

## Abstract

**Abstract:**

Clinical reasoning for acute dyspnoea: Comparison of final-year medical students from discipline- and competency-based undergraduate programmes.

**Background:**

The global shift to competency-based medical education aims to improve the performance of its trainees, including in the key competency domain of clinical reasoning. However, research on whether such education actually improves clinical reasoning is sparse. The purpose of this study is to compare assessed clinical reasoning performance in digitally presented cases of acute dyspnoea between final-year medical students from a traditional, discipline-based and those from an integrated, competency-based undergraduate programme.

**Methods:**

A total of 60 medical students in their final-year clerkships participated in the study; 30 were from a discipline-based programme, and 30 were from a competency-based programme of the same faculty. The students completed a knowledge test consisting of 22 single choice items and a computer-based test of clinical reasoning with six video-based case scenarios with different underlying diseases leading to dyspnoea. The operationalized measures of clinical reasoning were the number and relevance of the diagnostic tests chosen, time to diagnosis and diagnostic accuracy.

**Results:**

The two groups did not differ in their knowledge of the acute dyspnoea content domain. With regard to clinical reasoning, the selection of relevant tests, time required to make a diagnosis and accuracy of the diagnosis varied across the six case scenarios in both groups. However, the results from the measures of the clinical reasoning process did not differ between the students from the two types of undergraduate medical programmes. No significant differences were found with regard to the selection of relevant diagnostic tests (M = 63.8% vs. M = 62.8%), the time to a diagnosis (M = 128.7 s vs. M = 136.4 s) or the accuracy of diagnosis (M = 82.2% vs. M = 77.0%).

**Conclusions:**

Key indicators of the clinical reasoning process, when assessed with objectively measured parameters, did not differ between final-year medical students from a traditional, discipline-based and those from an integrated, competency-based undergraduate programme in the domain of acute dyspnoea. The results substantiate and expand those of previous studies based on subjective assessor ratings that showed limited change in the clinical reasoning performance of medical students with competency-based undergraduate education.

## Background

Undergraduate medical education, like the entire medical training continuum, is currently in a transition towards competency-based frameworks and curricula [1, 2]. Many medical faculties have reformed their undergraduate programmes from having traditional, discipline-based to integrated, competency-based curricula. Clinical reasoning represents a key competency for every physician and central outcome in competency-based training [3, 4] However, little empirical evidence exists on the extent to which competency-based education may improve the clinical reasoning ability of medical students. The purpose of the present study is to compare the clinical reasoning performance between final-year medical students from a discipline-based undergraduate programme and a competency-based undergraduate programme using a computer-based assessment instrument.

Clinical reasoning is an essential competency for every physician and serves as a foundation for the effective and safe execution of numerous medical tasks [3, 4]. It represents a complex cognitive process that commonly begins with patient presentation and results in a decision on an underlying diagnosis and/or the therapeutic approach to be taken. Early attempts to reform traditional discipline-based medical programmes placed the problem-based learning (PBL) teaching format at the centre of the curricular structure [5]. PBL is, to some degree, aligned with medical decision-making process, but it does not explicitly teach clinical reasoning in the decision-making process. Medical education reform has moved on, and the transition towards competency-based programmes currently represents the major direction for the entire medical education continuum, including undergraduate medical training [1, 2]. In today’s frameworks for competency-based medical education, clinical reasoning represents a key competency to be conveyed during training. These frameworks include, e.g., CanMEDS in Canada [6], the Accreditation Council for Graduate Medical Education (ACGME) in the USA [7], Outcomes for Graduates in the UK [8] and the National Catalogue of Learning Outcomes in Medicine (NKLM) in Germany [9].

Competency-based programmes differ from traditional, discipline-based medical degree programmes in that they are usually horizontally and vertically integrated, and the approach to curriculum design is centred around predefined outcomes to be met by the programme [10]. Furthermore, they generally offer early patient-based learning in clinical workplaces, supported by structured training in communication skills with simulated patients. It is generally assumed that the competency-based reorientation of medical education will lead to better trained physicians and thus to better care of patients [6, 11]. However, the results presented in the literature are limited and inconsistent regarding overall improvement and sparse regarding changes in clinical reasoning ability. The limited results and research related to effects of curricular change contrast the large number of medical schools that have undergone major programme reform in recent decades. In general, a number of studies and overviews have compared discipline-based with problem-based programmes [6, 12–17], but there have been only a few comparative studies of competency-based curricula. The results on competency-based curricula are ambiguous. From a broad perspective, Kerijk et al. [18] found no overall differences in acquired knowledge, performance in a clinical setting or preparedness for working as a physician in students graduating from the two types of programmes of the same institution. In another study, Dutch students in their final year of an integrated curriculum were compared with German students with a discipline-based curriculum in a setting simulating tasks on their first day as physician in a clinical workplace [19, 20]. Medical supervisors subjectively rated the students on Likert-based scales and gave higher ratings to students from the integrated, competency-based programme for the “active professional development” competency and the “solving a management problem” professional activity. In contrast, students from the discipline-based course received higher ratings in the area of “breaking bad news”. In the same study, when examining clinical decision-making more specifically, the educational researchers found no differences in supervisors’ subjective, semi-quantitative ratings for “clinical reasoning under time-pressure” between the final-year students from the discipline-based and competency-based programmes [20]. These findings are consistent with those of another study by this group in which supervising physicians from Utrecht and Hamburg rated the competencies of their graduates semi-quantitatively on a Likert scale [21]. Their ratings showed no differences between the two groups in skills of “solving medical problems” and “ability to prioritize tasks”, both of which can be attributed to the area of clinical reasoning.

With the advance of technology, new opportunities have emerged to assess the clinical reasoning performance of medical trainees. Technology-based testing allows objective and more differentiated measurements compared to the subjective Likert scale-based ratings that have been employed by supervisors. The Assessing Clinical Reasoning (ASCLIRE) test represents a feasible, effective and well-researched computer-based instrument for the assessment of clinical reasoning [4, 22–24]. The test is based on digitally presented cases of patients with acute dyspnoea and different underlying diagnoses. The test allows the evaluation of three partly correlated but distinct aspects of the clinical decision process: choice of relevant diagnostic information, time to a diagnostic decision and accuracy of the diagnostic decision. The ASCLIRE test mimics the clinical reasoning process to an appropriate degree with data acquisition, data interpretation, and data synthesis. Previous internal and external validation studies have shown that the test results i) have good psychometric properties; (ii) differentiate between trainees in different years of study; (iii) differentiate between experts and trainees; and (iv) have three separable latent factors in clinical reasoning, i.e., choice of relevant diagnostic information, decision time and diagnostic accuracy [4, 22–24].

The aim of this study is to compare the clinical reasoning performance of medical students in their final-year clerkships from a discipline-based or competency-based undergraduate medical programme. The clinical reasoning performance is assessed by using the ASCLIRE test and refers to the choice of relevant diagnostic information, decision time and diagnostic accuracy in digital cases of patients with various forms of acute dyspnoea. For this study, we took advantage of the brief opportunity at our medical faculty to have medical students in their final-year clerkships from both traditional, discipline-based and integrated, competency-based medical programmes.

## Methods

### Setting

The study was carried out in 2016 at the Charité – Universitätsmedizin Berlin (Charité). The study protocol was approved by the Charité data protection officer and the Charité ethics board (No. EA4–096-16).

#### Undergraduate medical programmes at the Charité

Approximately 300 new students are enrolled twice yearly in the first semester of the undergraduate medical programme at the Charité. Until the Summer 2010 term, students were enrolled in a traditional, discipline-based curriculum (TC), and beginning in the Winter 2010/11 term, they were enrolled in an integrated, competency-based curriculum (CC) [10]. Both programmes comprise twelve semesters and a total of 5500 teaching hours. In both programmes, students complete a final-year clerkship in the sixth year of study. This clerkship is divided into three trimesters with rotations in internal medicine, surgery and one elective discipline [10].

The traditional, discipline-based medical curriculum at the Charité (regular curriculum of medicine) is structured as a two-year pre-clinical section covering basic science subjects and a three-year clinical study section covering clinical science subjects [10]. The teaching formats in the pre-clinical section include lectures, seminars and practical sessions, while in the clinical section, there are lectures, seminars, simulations and bedside teaching. The symptoms and potential underlying diseases of dyspnoea are taught in semesters 5 to 10 through lectures, seminars, emergency simulations and bedside teaching.

The integrated, competency-based curriculum at the Charité (modular curriculum of medicine) was planned and implemented according to an outcome-oriented approach based on a framework for medical competencies developed at the Charité [10], which is comparable to the CanMEDS framework [6]. The programme is structured with 40 themed modules that integrate basic and clinical subjects throughout the programme. At the core of the longitudinal curriculum structure is clinical skills training bedside teaching with real patients, beginning in the first week of study, as well as the gradual increase in professional activities over the semester. Throughout the course, students are trained in communication skills for physicians and have weekly PBL sessions. The symptoms of dyspnoea and potential underlying diseases are taught in semesters 1 to 10 through lectures, seminars, PBL, emergency simulations and bedside teaching.

#### Design

To compare the two undergraduate programmes, we purposively selected medical students in the final-year clerkship. Students in this phase of study were chosen because, first, this phase is close to the end of programme, thus allowing testing to assess overall programme outcomes. Second, the preceding, mostly theoretical and classroom-based phases of study, both in the discipline-based and competency-based programmes, can be seen as completed and self-contained. The study was carried out at a point when the two programmes were running in parallel and it was possible to recruit final-year medical students from both programmes [25]. The data were collected on two test dates (T1: June 2016, only TC participants; T2: December 2016, both TC and CC participants).

#### Recruitment procedure

To recruit the participants, medical students in the final-year clerkship at the Charité and connected teaching hospitals were invited via email to voluntarily participate in the study at the end of their second rotations. We purposely chose the end of the second rotation and omitted the third rotation because we expected an insufficient participation rate due to the final state examination, which is taken after the third rotation. To reduce the effects of a long-term study, only students from the regular curriculum of medicine who had completed up to the 14th semester of their studies were recruited. The number of participants was restricted beforehand to 60 (a goal of *n* = 30 from each programme) due to the availability of resources (cost for computer licences, research staff, etc.). Altogether, 256 students from the regular curriculum of medicine and 229 students from the modular curriculum of medicine were contacted. The inclusion of participants in the study was based on the order of their replies and the fulfilment of the study criteria. Study participation was financially compensated.

#### Control variables

The participants signed the consent form and provided socio-demographic information, such as age, gender and semester. Because context-specific prior knowledge played an important role in comparing competency development, all participants participated in a knowledge test on the acute dyspnoea content domain. This test consisted of 22 single choice items, each with four choices. The test was conducted on paper, took approximately 30 min to complete and was undertaken after the clinical decision-making test.

#### Administration of the ASCLIRE test

The ASLIRE test consists of altogether six digital patients’ cases of acute dyspnea with different underlying disorder [4]. Each case begins with a short, video-based prototypical clinical presentation of a patient case. All clinical presentations are played by the same male standardized actor.

Following a general introduction and a training case, the participants worked individually on the randomly ordered cases of acute dyspnoea. For each case, the participants first watched the respective patient presentation video. Next, they were free to choose any type, order and number of diagnostic tests from a graphical interface of the computer screen. The test results were displayed via text (e.g., blood pressure), image (e.g., X-ray chest) or audio (e.g., heart rate) and had to be interpreted by the participants. For this part, the participants were instructed to work as quickly as possible to come to a decision regarding the underlying diagnosis without sacrificing accuracy. The participants could choose from 20 diagnoses available in a drop-down list on the computer screen. During the ASCLIRE testing, all selected diagnostic measures and the time required for all processes until a diagnosis was reached were recorded for each participant. The overall ASCLIRE test duration was approx. 60 min in total.

The clinical reasoning process was operationalized for each participant with three parameters: 1) relative proportion of the number and relevance of diagnostic measures (compared with those of the selections made by medical specialists in this field during the test validation); 2) diagnostic accuracy (correct or incorrect); and 3) time to diagnosis (in seconds from the end of the video presentation to the diagnostic decision).

#### Statistical analysis

The statistical analysis was carried out using SPSS 21 (IBM Deutschland GmbH, Ehningen, Germany). The data are presented as the mean and standard deviation. Results on correct or incorrect diagnostic accuracy are reported as the percentage of total answers given. Unpaired t-test were used to test for group differences in parametric variables. Chi-squared tests were applied to test for group differences in categorical variables. A *p* value lower than 0.05 was considered statistically significant. In the knowledge test, the number of correctly answered items was selected as the dependent variable. For the analysis of the ASCLIRE test, the following dependent variables were selected: 1) number and relevance of diagnostic measures; 2) time to diagnosis; and 3) diagnostic accuracy (correct or incorrect).

## Results

### Control variables

#### Socio-demographic variables

The comparison of the control variables revealed no significant difference in the age of participants (TC: M = 27.87 years, SD = 3.83; CC: M = 27.47 years, SD = 3.36 years). As expected, there was a small significant difference between the two groups in the number of semesters studied (t(37.33) = 3.33; *p* = 0.002 / TC: M = 12.87, SD = 1.28; CC: M = 12.03, SD = 0.49), which was mitigated as much as possible by limiting the number of semesters studied in the recruitment procedure.

#### Pre-existing knowledge variable

In the knowledge test on acute dyspnoea, there were no differences between the two groups in the number of correctly answered items (TC: M = 13.7 (62.1%), SD = 1.8; / CC: M = 13.6 (61.7%), SD = 2.1). On average, students answered 61.9% of items correctly, while the t-test showed no significant differences between the groups.

Overall, the distribution of the socio-demographic characteristics and domain-specific knowledge test scores indicated that the two cohorts were acceptably comparable and that we could continue to compare their clinical reasoning performance on the ASCLIRE test.

### ASCLIRE test results for clinical reasoning

**Fig. 1:**
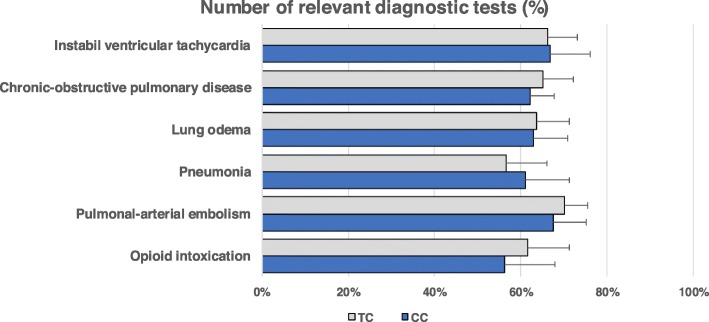
Number of relevant diagnostic tests chosen for six cases of acute dyspnoea with different underlying diseases by medical students in the final-year clerkship. Students were either from a traditional, discipline-based undergraduate medical programme (TC, *n* = 30) or an integrated, competency-based undergraduate medical programme (CC, *n* = 30) of the same medical faculty. The number of relevant diagnostic tests is based on a comparison with the selections made by medical specialists

**Fig. 2:**
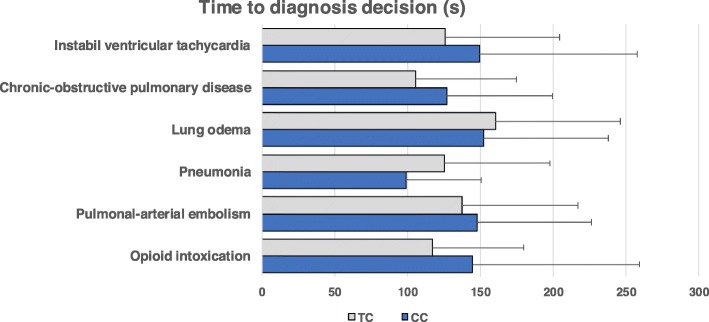
Time required to decide on an underlying diagnosis for six cases of acute dyspnoea with different underlying diseases by medical students in the final-year clerkship. Students were either from a traditional, discipline-based programme (TC, *n* = 30) or an integrated, competency-based undergraduate medical programme (CC, *n* = 30) of the same medical faculty. The time required to decide on a diagnosis in seconds refers to the time from the end of the case video presentation to the time of the diagnostic decision

**Fig. 3:**
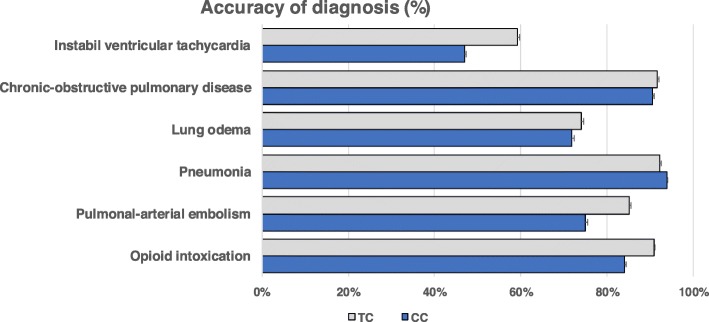
Choice of the correct diagnosis for six cases of acute dyspnoea with different underlying diseases by medical students in the final-year clerkship. Students were either from a traditional, discipline-based undergraduate medical programme (TC, *n* = 30) or an integrated, competency-based undergraduate medical programme (CC, *n* = 30) of the same medical faculty. Choice of the correct diagnosis was operationalized dichotomously as either correct or incorrect

In the two study cohorts, the following results were obtained through the administration of the ASCLIRE test to the final-year clerkship students:

1. **Selection of relevant diagnostic tests:** The number of chosen relevant diagnostic tests varied across all students (Fig. 1), with the highest number of tests chosen for the case of pulmonary artery embolism and the lowest number chosen for the case of opioid intoxication in comparison to the expert standard. Overall, the TC students selected, on average, 63.8% of relevant diagnostic tests. The CC students did not show a significantly different result (62.8%) for any of the six cases (numeric details in appendix 1).

(2) **Time required to decide on a diagnosis:** There was considerable variation in the time to diagnostic decision between the cases (Fig. 2). The mean time was longest for the case of lung oedema and shortest for the case of pneumonia. While TC students needed an average of 128.7 s across all cases to decide on a diagnosis, CC students needed 136.4 s. The difference was not significant for any of the six cases (numeric details in appendix 1).

(3) **Choice of the correct diagnosis:** The accuracy of the chosen diagnosis varied across all students (Fig. 3), with the highest accuracy for the case of pneumonia and the lowest accuracy for the case of unstable ventricular tachycardia in comparison to the expert standard. Overall, the accuracy in choosing the correct diagnosis was an average of 82.2% for TC students and 77.0%. for CC students. The difference was not significant for any of the six cases (numeric details in appendix 1).

## Discussion

Many medical schools have undergone major curriculum reform in recent decades, but little research comparing the performances of students from previous and new programmes is available. Those who have been managing themselves a major curriculum reform may not be surprised by this discrepancy. Similar to us, the authors of this articles, they likely have experienced that the implementation of a new programme and the parallel major organizational changes exhaust most if not all resources that can be mobilized to make the curricular change happen. In addition, they may have recognized that there are few opportunities to carry out such comparative studies in a feasible, reliable and valid manner. The following two examples may illustrate this difficulty. At our institution, we considered comparing the results of a large written final state exam with 320 multiple choice questions (MCQs) to potentially detect the differences in the medical knowledge acquired in the two programmes. However, we refrained from conducting this investigation, as most students of both study programmes learn specifically for the final state exam using the same commercially available platform that provides specific training based on the MCQs used in previous exams. Thus, the results on acquired knowledge would potentially be biased by the phenomenon of learning for the test using the same learning materials and MCQs. Next, we considered holding a comparative objective structured clinical examination (OSCE) to potentially detect differences in the practical skills acquired in the two programmes. However, again, we refrained from doing so, as the traditional curriculum does not include OSCE assessments, while OSCE assessments have a prominent role in the competency-based programme. Thus, the results for practical skills would have potentially been biased by the familiarity of the CC students with the test format [26]. In the following section, we will discuss the results of this study first in the context of research using the ASCLIRE test in general and second in the context of comparing clinical reasoning performance between different undergraduate medical programmes in particular.

The overall results we found with the ASCLIRE test for the final-year medical students in our study fit well and complemented the previous findings reported by Kunina-Habenicht et al. [4] on test participants with different levels of expertise. Across all six cases, the participants’ results for choosing the correct diagnosis (TC students 82%, CC students 77%) ranked between those of medical experts (94%) and those of medical students before entering the final-year clerkship (56%, study years 1–5). The same finding was observed for the time to a diagnostic decision: the TC students (129 s) and CC students (136 s) ranked between medical experts (122 s) and medical students from years 1–5 (187 s). Thus, with increasing experience, the number of correct diagnoses increases, while the time to come to a diagnostic decision decreases. The consistent findings on student’s rankings add further to the validity evidence for the ASCLIRE test in general but as well as to the validity evidence for the results of this study.

When examining the results of our study comparing the two study programmes, we were surprised to find no significant differences in the clinical reasoning performances between the final-year clerkship students from the traditional discipline-based and integrated, competency-based medical programmes of our medical faculty. We had expected that the students of the competency-based medical programme would perform better given the early patient exposure, longitudinal PBL, communication and skills training and explicit “medical decision-making” outcome in the new programme. However, with the objective assessment, we found no significant difference in the selection of relevant tests, the time to diagnosis or the choice of diagnosis in the acute dyspnoea domain. This finding was robustly demonstrated across all six cases of acute dyspnoea. Importantly, regarding the interpretation of our finding, the level of knowledge in the domain of acute dyspnoea did not differ between the two groups.

Overall, our study results complement the research on the influence of competency-based medical education on clinical decision-making ability. It expands and substantiates the of reports by Wijnen-Meijer et al. [19–21], who utilized subjective evaluations by employing objectively measurable parameters for clinical decision-making. Second, our study was conducted at one medical faculty in one country, which eliminates the potential impact of studying in different countries or medical schools due to context-specific practices and attitudes. On the other hand, the lack of differences in the acute dyspnoea content domain should not be generalized to assume that there are no differences in clinical reasoning in general or in other content domains. Clinical reasoning represents a complex problem-solving task that is strongly context-dependent [27]. The research in this area is limited by the availability of assessment instruments. The ASCLIRE test is available for this specific content domain but not for other domains that would allow a broader range of testing of clinical decision-making in other content domains. We considered the results of this study as a small but important piece in the puzzle of the overall picture of the impact of competency-based education on the clinical reasoning of medical students. With this report, we aim to make these so-called “negative results” available to the community to stimulate further research in this area and to make them accessible for potential future meta-analysis comparing the performance of medical students from competency-based programmes with that of medical students from other types of programmes.

Beyond these general considerations for the interpretation of our study results, the following particular conditions may have impacted the outcome of our study. First, the management of patients with acute dyspnoea was well covered in both types of undergraduate medical programmes, as it likely is in most, if not all, undergraduate medical programmes. Second, clinical decision-making was not explicitly taught and trained in either programme. It may be necessary to include formats that explicitly convey and train clinical decision-making to actually improve it.

This study has limitations beyond those discussed above. It was a single-centre study, which may limit the extent to which it is transferable to other contexts. Participants were not selected randomly. Due to the limited number of participants, it is not certain whether the sample represents the entire cohort. It would have been preferable to include whole annual cohorts from both undergraduate medical programmes and to test clinical decision-making in several content domains. This approach was prevented because it was not possible to implement new obligatory assessments for research purposes.

In conclusion, in the acute dyspnoea content domain, we found no differences in three objectively measurable parameters for clinical decision-making between medical students from a discipline-based and a competency-based programme. These results are consistent with subjective ratings by medical supervisors on clinical decision-making [19–21]. This study is intended to stimulate more and broader research on actual clinical reasoning performance in current competency-based undergraduate medical education programmes.

## Supplementary information

**Additional file 1.**

## Data Availability

The dataset used and analysed during this study is available from the corresponding author on request where warranted.

## Abbreviations

ACGME: Accreditation Council for Graduate Medical Education
ASCLIRE: Computer-based test for assessing clinical reasoning
CC: Integrated, competency-based curriculum
NKLM: National Catalogue of Learning Outcomes in Medicine
OSCE: Objective structured clinical exams
PBL: Problem-based learning
TC: Traditional, discipline-based curriculum
