# Supplementary material for: Clinical reasoning for acute dyspnoea: comparison between final-year medical students from discipline- and competency-based undergraduate programmes
Source: BMC Med Educ. 2020 May 19;20:161. doi: 10.1186/s12909-020-02055-y (PMC7238574; doi:10.1186/s12909-020-02055-y)
Supplement: Supplementary file 1 — Additional file 1. [file 12909_2020_2055_MOESM1_ESM.pdf]

Additional file 1 - DOI: 10.1186/s12909-020-02055-y

Clinical reasoning for acute dyspnoea: comparison between final-year medical students from discipline- and competency-based undergraduate programmes

1. Number of relevant diagnostic tests (%)

Case	Curriculum	Mean	Standard Deviation	T	p
**1**	TC	66.231	6.826	-0.130	0.897
	CC	66.513	9.450		
**2**	TC	65.099	7.124	1.868	0.067
	CC	61.914	5.734		
**3**	TC	63.650	7.521	0.299	0.766
	CC	63.021	8.273		
**4**	TC	56.742	9.390	-1.064	0.294
	CC	61.133	10.594		
**5**	TC	70.198	5.317	1.536	0.130
	CC	67.457	7.775		
**6**	TC	61.721	9.487	1.839	0.068
	CC	55.337	10.192		

2. Time to diagnostic decision (sec)

Case	Curriculum	Mean	Standard Deviation	T	p
**1**	TC	126.17885	78.137192	-0.931	0.356
	CC	149.65387	108.000141		
**2**	TC	105.71885	69.007628	-1.125	0.265
	CC	126.89537	72.645262		
**3**	TC	160.36233	86.020105	0.368	0.751
	CC	152.17240	82104,488		
**4**	TC	125.32074	72.559985	1.546	0.129
	CC	99.27643	51.582800		
**5**	TC	137.46426	79.654570	-0.496	0.622
	CC	147.844,40	78.327660		
**6**	TC	117.40630	62.226271	-1.134	0.263
	CC	144.79757	114.951967		

3. Accuracy in diagnosis (%)

Case	Curriculum	Correct diagnose	Standard Deviation	X^2	p
**1**	TC	90.90	0.29	0.904	0.342
	CC	84,000	0.37		
**2**	TC	85.20	0,36	0.044	0.834
	CC	75.00	0.44		
**3**	TC	92.30	0.27	0.004	0.949
	CC	93.80	0.25		
**4**	TC	74.10	0.45	0.22	0,822
	CC	71.90	0.46		
**5**	TC	91.70	0.28	0.662	0.416
	CC	90.60	0.30		
**6**	TC	59.30	0.05	0.581	0.446
	CC	46.90	0.51		
